# Interaction between BDNF val66met polymorphism and personality on long-term cardiac outcomes in patients with acute coronary syndrome

**DOI:** 10.1371/journal.pone.0226802

**Published:** 2019-12-30

**Authors:** Jae-Min Kim, Robert Stewart, Seon-Young Kim, Ju-Wan Kim, Hee-Ju Kang, Ju-Yeon Lee, Sung-Wan Kim, Il-Seon Shin, Min Chul Kim, Young Joon Hong, Youngkeun Ahn, Myung Ho Jeong, Jin-Sang Yoon

**Affiliations:** 1 Departments of Psychiatry, Chonnam National University Medical School, Gwangju, Korea; 2 King’s College London, Institute of Psychiatry, Psychology and Neuroscience, London, United Kingdom, and South London and Maudsley NHS Foundation Trust, London, United Kingdom; 3 Department of Cardiology, Chonnam National University Medical School, Gwangju, Korea; National Institutes of Health, UNITED STATES

## Abstract

**Background:**

The prognostic role of BDNF val66met polymorphism on long-term cardiac outcomes in acute coronary syndrome (ACS) has been unclear. Environmental factors may modify the association, but these have not been investigated to date. This study aimed to investigate the potential interactive effects of BDNF val66met polymorphism and personality traits, one of the main environmental prognostic factors of ACS, on major adverse cardiac events (MACEs) in patients with ACS.

**Methods:**

A total of 611 patients with recent ACS were recruited at a university hospital in Korea. Baseline evaluations from 2007 to 2012 assessed BDNF val66met polymorphism and personality using the Big Five Inventory, which yielded two personality clusters (resilient and vulnerable) and five dimensions (extraversion, agreeableness, conscientiousness, neuroticism, and openness). Over a 5~12 year follow-up after the index ACS, times to MACE were investigated using Cox regression models after adjustment for a range of covariates.

**Results:**

The BDNF val66met polymorphism modified the associations between vulnerable personality type and worse long-term cardiac outcomes in ACS patients with significant interaction terms, in that the associations were statistically significant in the presence met allele. Similar findings were observed for the individual personality dimensions of agreeableness and neuroticism.

**Conclusions:**

Gene (BDNF val66met polymorphism) x environment (personality traits) interactions on long-term cardiac outcomes were found in ACS.

## Introduction

Acute coronary syndrome (ACS), including myocardial infarction (MI) and unstable angina (UA), is a global leading cause of disease burden associated with increased morbidity and mortality [1]. Considerable efforts have been made to elucidated the predictive factors for ACS prognosis [2,3]. Gene-environment interactions (GEI) in human diseases have been received increasing research attention [4]. However in cardiovascular diseases, most studies have focused on only lifestyle and nutritional factors as environmental factors [5]. Our study group has carried out a large cohort study of patients with ACS, the DEPression in ACS (DEPACS) study, to investigate the inter-relationships of both biological and psychosocial factors with ACS prognosis [6,7]. Using the data, we recently published the interactive effect of anxiety and serotonin transporter gene on long-term cardiac outcomes in ACS [8]. As far as we aware, this was the first GEI report considering the psychosocial one as an environmental factor for ACS prognosis, which might give promise of other GEIs in this research field.

In the present study, we focused on the interactive role of brain-derived neurotrophic factor (BDNF) gene and personality traits. The rationales for choosing BDNF gene were that serum BDNF plays a crucial role in cardiovascular disease by enhancing angiogenesis, regulating vascular flow, and promoting the revascularization of ischemic tissue [9,10]; and that BDNF expression is influenced by genetic polymorphisms, including a single-nucleotide polymorphism at nucleotide 196G/A, which results in the substitution of valine by methionine at codon 66 (val66met) of the pro-BDNF molecule, and thus presence of the *met* allele has been linked to decreased activity-dependent secretion of BDNF [11]. However, the associations between BDNF val66met polymorphism and ACS prognosis have been controversial. Some studies have found significant associations of the *met* allele with increased risk of MI in humans [12]; while other have reported no associations [13] or associations with better prognosis [14,15].

We speculated that personality traits might have influences on BDNF genetic sensitivity in ACS prognosis, and so give rise to those heterogeneous findings at least in part. These hypotheses were based on the previous findings on the associations of BDNF val66met polymorphism with several personality traits as well as with ACS prognosis [16–18]. Therefore, interactions between this polymorphism and personality traits on ACS prognosis might be expected but have not been investigated to date. Early personality studies on the associations with ACS prognosis have concentrated on type D personality, characterized by negative affectivity and social inhibition [19,20]. However, type D is a limited and specific aspect of personality, and currently the Five Factor Model (FFM) is a more accepted, widely used and comprehensive means of categorization [21]. The FFM consists of the following dimensions: extraversion, agreeableness, conscientiousness, neuroticism, and openness. However, the impacts of FFM personality dimensions on ACS prognosis have not been formally investigated yet.

This study therefore aimed to investigate the interactive effects of BDNF val66met polymorphism and FFM personality dimensions on long-term cardiac outcomes in a large and representative sample of patients with ACS.

## Methods

### Study outline and participants

**Fig 1:**
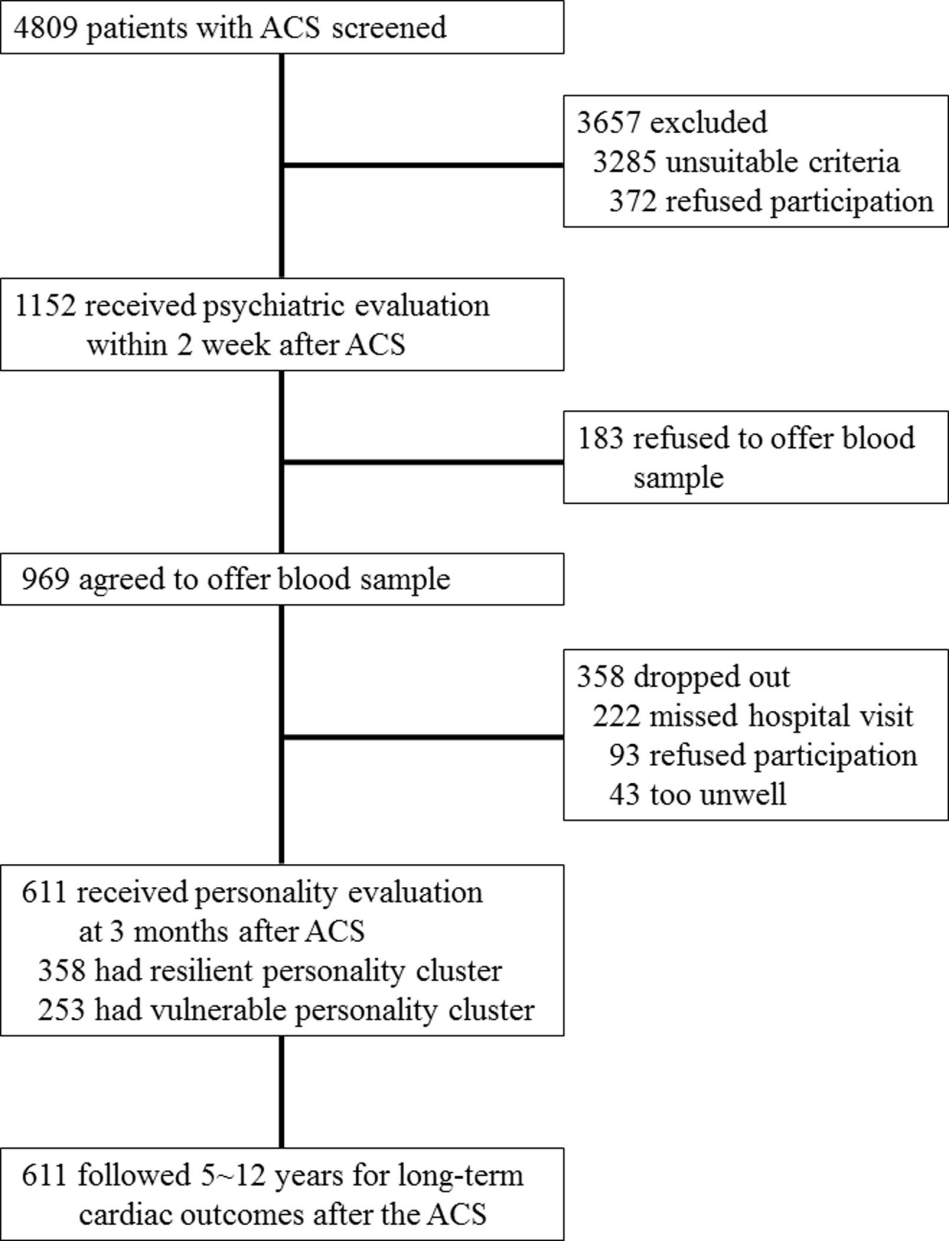
Flow chart. ACS: acute coronary syndrome, Baseline 969 participants, agreed to offer blood sample, received all psychiatric evaluations other than personality within 2 weeks after the index ACS episodes. Of them, 611 received personality evaluation at 3 months, and followed 5~12 years for cardiac outcomes after the ACS.

All analyses were carried out using data from the DEPACS study, which design and main findings have been published [6–8]. The outline and participants recruitment process for the present analysis are presented in Fig 1. Participants were consecutively recruited from patients recently hospitalized with ACS (N = 4809) at the Department of Cardiology of Chonnam National University Hospital, Gwangju, South Korea from 2006 to 2012. This Department was nominated by the Korean Circulation Society to serve as the central coordinating center for the Korea Acute Myocardial Infarction Registry (KAMIR) [22]. KAMIR is a nationwide prospective multicenter online registry (http://kamir5.kamir.or.kr/) designed as a surveillance platform to track clinical outcomes of patients with acute MI without exclusion criteria to reflect real-world practice; this enables prospective associations to be evaluated for a range of exposures or interventions with long-term cardiac outcomes. Patients who met eligibility criteria (detailed in online supplementary material) and who agreed to participate (N = 1152) were examined for baseline evaluations as inpatients within 2 weeks (mean 6.3 ± SD 2.4 days) post-ACS. Of these, 969 (84.1%) agreed to offer blood sample for genotyping. Personality was assessed 3 months after the baseline evaluation, and the 611 who received personality assessments formed the sample for the analyses presented here. All participants were followed for cardiac outcomes until 2017, 5~12 years after the index ACS. Written informed consent was obtained from each participant, and the study was approved by the CNUH Institutional Review Board.

### Baseline evaluations

Information at baseline was collected on characteristics that could potentially affect cardiac outcomes [23] and/or might differ by BDNF val66met polymorphism [15]. Demographic data on age, gender, education, marital status, living alone, accommodation, and employment status were obtained. For evaluating depressive symptoms, the self-completed Beck Depression Inventory (BDI) [24] was completed, as well as psychiatrist-administered Mini-International Neuropsychiatric Interview (MINI) [25] diagnoses covering depressive disorders listed in the Diagnostic and Statistical Manual of Mental Disorders, 4^th^ Edition (DSM-IV). Previous episodes of depression and family history of depression were also recorded. The following cardiovascular risk factors were ascertained: diagnosed hypertension and diabetes mellitus, hypercholesterolemia by fasting serum total cholesterol level (>200mg/dL), obesity by measured body mass index (BMI), reported current smoking status, and previous and family histories of ACS. Cardiac severity status was estimated on the basis of ACS diagnosis (MI or unstable angina), Killip classification [26], left ventricular ejection fraction (LVEF), and serum levels of troponin I, creatine kinase-MB (CK-MB) and high sensitivity C-reactive protein (hs-CRP). The BDNF polymorphism was ascertained (detailed in online supplementary material), and the genotype was categorized as val/val, val/met, or met/met.

### Personality assessments

Personality was assessed by the Big Five Inventory (BFI), which was selected to represent the core FFM personality traits [27]. Terminology applied to these five traits has been described as follows: ‘Extraversion’—talkative, assertive, and energetic; ‘Agreeableness’—good-natured, cooperative, altruistic and empathic; ‘Conscientiousness’—orderly, responsible, and dependable; ‘Neuroticism’—neurotic, easily upset and not self-confident; and ‘Openness’—openness to experience, intellectual, imaginative, and independent-minded. This is a self-reported measure filled out by participants. Scores are made on a scale from 1 to 5 for each of 44 scale items, and higher scores represent higher levels of each given trait [27]. The BFI has been formally translated and validated in Korean [28].Unfortunately a prospective design with pre-morbid personality was not possible in the present clinical setting; however, the BFI was performed 3 months after the baseline assessment to minimize the influence of the ACS and the reaction of patients to this diagnosis.

The FFM can be investigated either as five dimensions separately (a dimensional or variable-centered approach) or in combination (a typological or person-centered approach) [29,30]. For the typological approach, two personality clusters were identified from the results of identical analyses with the two-step procedure specified by Asendorpf et al. [31]. The personality profiles of the two clusters are shown in online S1 Fig, with z scores used for ease of interpretation. As in previous studies, the first cluster was labeled as resilient type and the second as vulnerable type [32,33]. Compared with the vulnerable type, the resilient type was characterized by significantly higher extraversion, higher agreeableness, higher conscientiousness, but lower neuroticism (all P-value<0.001). From a clinical perspective, the typological approach provides a more integrative framework for personality and health assessment, and thus has drawn more attention recently [30]. Therefore in this study, the personality cluster (resilient vs. vulnerable; typological approach) was considered as the primary exposure and the individual five personality dimensions (higher vs. lower scores for each; dimensional approach) were evaluated as additional exposure constructs.

### Long-term cardiac outcomes

Comprehensive evaluations for cardiac outcomes were possible for this study because the KAMIR manages and records detailed data on hospital admissions, deaths, recurrent MI, and percutaneous coronary intervention (PCI), electronically. Preplanned monitoring of the obtained information was conducted by additional personnel, with expertise in data quality control, and checked for completeness and accuracy every one year from the registration. All missing or uncertain outcome data were complemented or amended through manual checks of electronic records and supplementary phone calls where required. This allow complete follow-up for all baseline participants. To enable non-hierarchic endpoint analyses, all patients were followed for the evaluation point of interest or until death. The primary endpoint was a major adverse cardiac event (MACE), which was a composite of all-cause mortality, MI, and PCI. Secondary endpoints were all-cause mortality, cardiac death (defined as sudden death when no other explanation was available, death from arrhythmias or after MI or heart failure, or death caused by heart surgery or endocarditis), MI, and PCI. An independent endpoint committee composed of study cardiologists adjudicated all potential events and was blinded to participants’ depression comorbidity.

### Statistical analysis

Demographic and clinical characteristics at baseline were compared between the two personality clusters (resilient vs. vulnerable) by t-tests or χ2 tests as appropriate. Hardy-Weinberg equilibrium was tested by using the Haploview v.4.2 program. Characteristics significantly associated with personality cluster (P<0.05) and other variables with potential effects on MACE or potentially differing by BDNF val66met polymorphism [15,23] were used as covariates in further adjusted analyses. Individual effects of personality cluster and BDNF val66met polymorphism on time to first MACE were estimated by Cox proportional hazards models before and after adjustment for potential covariates. Interactive effects of personality cluster and BDNF val66met polymorphism on time to first composite MACE were investigated in the same Cox proportional hazards models, as well as stratifying the associations of personality with composite MACE by BDNF val66met polymorphism. The same statistical analyses were carried out using individual five personality dimensions additionally and described in the online supplement material. Additional sensitivity analyses were conducted after excluding those who taking antidepressants a one year follow-up. All statistical tests were two-sided with a significance level of 0.05. Statistical analyses were carried out using SPSS 21.0 and STATA 12.0 software.

## Results

### Recruitment and baseline characteristics

Of the 1,152 participants in the baseline sample, data on genotype and personality were available for 611 (53%). Compared with the remainder, those included in this analysis were younger (P-value = 0.018), had higher BDI score (P-value<0.001), hypercholesterolemia (P-value = 0.006), and lower Killip class (P-value = 0.012). Baseline characteristics are presented in the 1^st^ column of Table 1. Vulnerable personality type was present in 253 (38.5%) of the 611 analysed participants. Baseline characteristics were compared between those with resilient and vulnerable personality type and presented in the 2^nd^ ~ 5^th^ columns of Table 1. The vulnerable group was more likely to be living in rented accommodation, and to report a previous history of depression, ACS, and hypertension, as well as having higher BDI scores and being more likely to have a major depressive disorder diagnosis. These characteristics and all other variables on cardiac risk factors and current cardiac status, which have been associated with cardiac outcomes or potentially differing by BDNF val66met polymorphism in previous researches [15,23], were included as covariates in later adjusted analyses.

**Table 1:** Baseline characteristics according to personality type in 611 patients with acute coronary syndrome (ACS).

	All (N = 611)	Resilient (N = 358)	Vulnerable (N = 253)	Statistical coefficient	P-value*
**Sociodemographic characteristics**					
Age, mean (SD) years	57.7 (10.9)	58.1 (10.7)	57.1 (11.1)	t = +1.071	0.284
Gender, N (%) female	173 (28.3)	93 (26.0)	80 (31.6)	χ^2^ = 2.326	0.127
Education, mean (SD) years	9.9 (4.5)	9.7 (4.6)	10.3 (4.3)	t = -1.575	0.116
Unmarried marital status, N (%)	80 (13.1)	50 (14.0)	30 (11.9)	χ^2^ = 0.579	0.447
Living alone, N (%)	53 (8.7)	33 (9.2)	20 (7.9)	χ^2^ = 0.322	0.570
Housing, N (%) rented	108 (17.7)	52 (14.5)	56 (22.1)	χ^2^ = 5.898	0.015
Currently unemployed, N (%)	219 (35.8)	126 (35.2)	93 (36.8)	χ^2^ = 0.158	0.691
**Depression characteristics**, N (%)					
BDI, mean (SD) score	11.0 (8.7)	8.7 (7.8)	14.4 (9.0)	t = -8.357	<0.001
DSM-IV depressive disorders					
No depressive disorder	341 (55.8)	245 (68.4)	96 (37.9)		
Minor depressive disorder	139 (22.7)	56 (15.6)	83 (32.8)	χ^2^ = 56.171	<0.001
Major depressive disorder	131 (21.4)	57 (15.9)	74 (29.2)		
Previous depression	26 (4.3)	10 (2.8)	16 (6.3)	χ^2^ = 4.536	0.033
Family history of depression	17 (2.8)	8 (2.2)	9 (3.6)	χ^2^ = 0.959	0.327
**Cardiac risk factors**, N (%)					
Hypertension	288 (47.1)	153 (42.7)	135 (53.4)	χ^2^ = 6.712	0.010
Diabetes mellitus	134 (21.9)	75 (20.9)	59 (23.3)	χ^2^ = 0.486	0.485
Hypercholesterolemia	325 (53.2)	191 (53.4)	134 (53.0)	χ^2^ = 0.009	0.925
Obesity	263 (43.0)	151 (42.2)	112 (44.3)	χ^2^ = 0.264	0.607
Current smoker	242 (39.6)	143 (39.9)	99 (39.1)	χ^2^ = 0.041	0.839
Previous ACS	30 (4.9)	11 (3.1)	19 (7.5)	χ^2^ = 6.251	0.012
Family history of ACS	19 (3.1)	10 (2.8)	9 (3.6)	χ^2^ = 0.287	0.592
**Current cardiac status**					
ACS diagnosis, N (%)					
Myocardial infarction	448 (73.3)	265 (74.0)	183 (72.3)	χ^2^ = 0.271	0.642
Unstable angina	163 (26.7)	93 (26.0)	70 (27.7)		
Killip class >1, N (%)	93 (15.2)	53 (14.8)	40 (15.8)	χ^2^ = 0.116	0.733
LVEF, mean (SD) %	60.8 (11.2)	60.3 (11.4)	61.4 (10.9)	t = -1.128	0.260
Troponin I, mean (SD) mg/dL	9.6 (14.2)	9.1 (13.8)	10.4 (14.7)	t = -1.085	0.278
CK-MB, mean (SD) mg/dL	17.3 (37.8)	16.9 (35.2)	17.7 (41.3)	t = -0.253	0.800
Hs-CRP, mean (SD) mg/L	1.03 (1.8)	1.04 (2.1)	1.01 (1.46)	t = +0.176	0.861

*p-values using t-tests or χ^2^ tests as appropriate.

BDI: Beck Depression Inventory; DSM-IV: Diagnostic and Statistical Manual of Mental Disorders, 4^th^ edition; LVEF: left ventricular ejection fraction; CK-MB: Creatine kinase-MB; hs-CRP: high sensitivity C-reactive protein.

### Individual associations between BDNF val66met polymorphism and MACE outcomes

Participants were followed for 5~12 years up to 2017 or until they died [median; mean (SD) duration of follow-up = 8.5; 8.8 (1.5) years] and the primary endpoint (composite MACE) was ascertained to have occurred in 259 (42.4%) by the end of this period. Numbers with secondary endpoints were: 106 (17.3%) for all-cause mortality; 63 (10.3%) for cardiac death; 82 (13.4%) for MI; and 103 (16.9%) for PCI. With respect to genotype frequency, the BDNF val/val, val/met, and met/met polymorphisms were present in 151 (24.7%), 324 (53.0%), and 136 (22.3%) patients, respectively. The distribution of the genotype did not deviate from the Hardy-Weinberg equilibrium (P-value = 0.13). The individual associations of BDNF val66met polymorphism with MACE outcomes are summarized in online S1 Table. No significant associations were found between the polymorphism and any MACE outcome before and after adjustment for age, gender, education, accommodation, Beck Depression Inventory scores, previous history of depression, hypertension, diabetes, hypercholesterolemia, obesity, smoking, past history of ACS, ACS diagnosis, Killip class, left ventricular ejection fraction, and serum levels on troponin I, creatine kinase-MB, and hs-CRP at baseline.

### Interactions of BDNF val66met polymorphism and personality cluster on composite MACE

**Fig 2:**
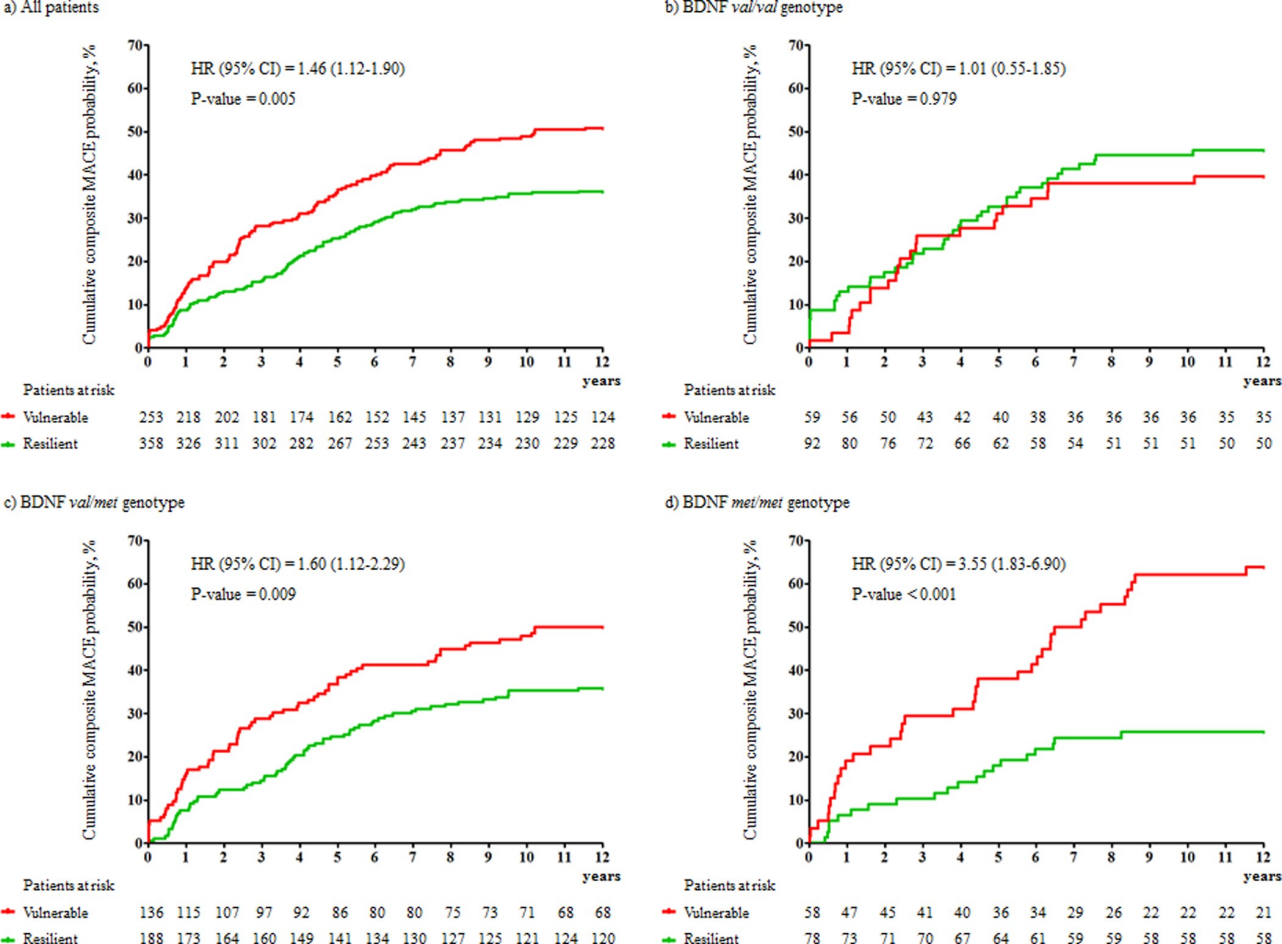
Associations of personality cluster with cumulative incidence (%) of composite major adverse cardiac events (MACE) by brain derived neurotrophic factor (BDNF) val66met polymorphism. Hazard ratios (95% confidence intervals) [HR (95% CI)] using Cox proportional hazards models were calculated after adjustment for age, gender, education, accommodation, Beck Depression Inventory scores, previous history of depression, hypertension, diabetes, hypercholesterolemia, obesity, smoking, past history of ACS, ACS diagnosis, Killip class, left ventricular ejection fraction, and serum levels on troponin I, creatine kinase-MB, and high sensitivity C-reactive protein at baseline. Multiplicative interaction terms between personality cluster and BDNF val66met polymorphism on composite MACE were statistically significant (P-value = 0.001). Figure a) denotes the association between personality cluster and composite MACE in all participants, and other Figures denote those by BDNF polymorphism: b) val/val, c) val/met, and d) met/met genotypes.

Associations of personality cluster with occurrence of composite MACE in all participants and by BDNF val66met polymorphism are presented visually in Fig 2. Vulnerable personality type was significantly associated with composite MACE in all participants in the same adjustment model. Stratifyed by BDNF polymorphism, the association between vulnerable personality type and composite MACE was not significant in patients with the val/val polymorphism, but was statistically significant and increased incrementally in strength with increased numbers of *met* alleles. The multiplicative interaction term between BDNF val66met polymorphism and vulnerable personality type on composite MACE was statistically significant (P-value = 0.001). Antidepressants were being taken by 12 participants (5 resilient and 7 vulnerable personality type) at the 1 year follow-up point. When the same analyses were repeated after excluding these participants, the results were not changed substantially.

### Interactions of BDNF val66met polymorphism and personality cluster on individual MACE components

Interactive effects on individual MACE outcomes are summarized in Table 2. In the full analysed sample, vulnerable personality type was significantly associated with PCI but not with other individual MACE outcomes. Stratified by BDNF val66met polymorphism, associations of vulnerable personality type with all-cause mortality were statistically significant only in the presence of met/met genotype; and those with PCI were statistically significantly in the presence of the *met* allele. However, multiplicative interaction terms with genotype were significant only for PCI (P-value = 0.024).

**Table 2:** Effect of personality cluster on cumulative incidence (%) of individual cardiac outcomes by brain derived neurotrophic factor (BDNF) val66met polymorphism.

Event	Patients group	Personality cluster	N patients	N (%) events	Unadjusted HR (95% CI)	Adusted		P-value for interaction^b^
						HR (95% CI)	P-value^a^	
All-cause mortality	All patients	Resilient	358	53 (14.8)	Ref	Ref	0.184	
		Vulnerable	253	53 (20.9)	1.47 (1.00–2.15)	1.32 (0.88–2.00)		
	**BDNF**							
	*val/val*	Resilient	92	17 (18.5)	Ref	Ref	0.372	0.262
		Vulnerable	59	10 (16.9)	0.93 (0.42–2.02)	0.64 (0.24–1.70)		
	*val/met*	Resilient	188	29 (15.4)	Ref	Ref	0.203	
		Vulnerable	136	31 (22.8)	1.56 (0.94–2.58)	1.43 (0.83–2.48)		
	*met/met*	Resilient	78	7 (9.0)	Ref	Ref	0.026	
		Vulnerable	58	12 (20.7)	2.41 (0.95–6.11)	3.65(1.17–11.42)		
Cardiac death	All patients	Resilient	358	33 (9.2)	Ref	Ref	0.580	
		Vulnerable	253	30 (11.9)	1.33 (0.81–2.18)	1.16 (0.68–1.98)		
	**BDNF**							
	*val/val*	Resilient	92	9 (9.8)	Ref	Ref	0.324	0.884
		Vulnerable	59	6 (10.2)	1.05 (0.37–2.94)	0.50 (0.13–1.98)		
	*val/met*	Resilient	188	18 (9.6)	Ref	Ref	0.387	
		Vulnerable	136	18 (13.2)	1.45 (0.76–2.80)	1.38 (0.67–2.86)		
	*met/met*	Resilient	78	6 (7.7)	Ref	Ref	0.203	
		Vulnerable	58	6 (10.3)	1.39 (0.45–4.31)	2.72(0.58–12.63)		
Myocardial infarction	All patients	Resilient	358	41 (11.5)	Ref	Ref		
		Vulnerable	253	41 (16.2)	1.53 (0.99–2.36)	1.34 (0.84–2.13)	0.218	
	**BDNF**							
	*val/val*	Resilient	92	11 (12.0)	Ref	Ref		0.150
		Vulnerable	59	6 (10.2)	0.83 (0.32–2.33)	0.89 (0.24–3.35)	0.865	
	*val/met*	Resilient	188	23 (12.2)	Ref	Ref		
		Vulnerable	136	21 (15.4)	1.38 (0.76–2.49)	1.42 (0.75–2.67)	0.281	
	*met/met*	Resilient	78	7 (9.0)	Ref	Ref		
		Vulnerable	58	14 (24.1)	2.92 (1.16–7.33)	2.50 (0.87–7.20)	0.089	
Percutaneous coronary intervention	All patients	Resilient	358	46 (12.8)	Ref	Ref		
		Vulnerable	253	57 (22.5)	1.94 (1.32–2.87)	1.82 (1.19–2.77)	0.005	
	BDNF							
	*val/val*	Resilient	92	15 (16.3)	Ref	Ref		0.024
		Vulnerable	59	10 (16.9)	1.06 (0.48–2.37)	0.84 (0.48–1.95)	0.227	
	*val/met*	Resilient	188	24 (12.8)	Ref	Ref		
		Vulnerable	136	32 (23.5)	2.11 (1.24–3.58)	2.21 (1.25–3.90)	0.006	
	*met/met*	Resilient	78	7 (9.0)	Ref	Ref		
		Vulnerable	58	15 (25.9)	3.21 (1.31–7.87)	3.95 (1.21–12.93)	0.023	

HR (95% CI) were calculated using Cox proportional hazards models.

^a^Adjusted for age, gender, education, accommodation, Beck Depression Inventory scores, previous history of depression, hypertension, diabetes, hypercholesterolemia, obesity, smoking, past history of ACS, ACS diagnosis, Killip class, left ventricular ejection fraction, and serum levels on troponin I, creatine kinase-MB, and high sensitivity C-reactive protein at baseline.

^b^Multiplicative interaction terms between personality cluster and BDNF val66met polymorphism on cardiac outcomes in the same adjusted model.

### Interactions of BDNF val66met polymorphism and individual personality dimensions on MACE

Interactive effects of BDNF val66met polymorphism and individual personality dimensions are summarized in in S2–S6 Tables. In general, the strengths of the association increased incrementally with increased numbers of *met* alleles, as described for the associations of personality cluster with composite MACE and its individual components. In particular, the associations of lower agreeableness with composite MACE were statistically significant only in the presence of met/met genotype, and associations of higher neuroticism with composite MACE were statistically significant in the presence of the *met* allele. Multiplicative interaction terms were significant for lower agreeableness and higher neuroticism on composite MACE. In addition, associations of lower extraversion with PCI were only statistically significant in the presence of the *met* allele; associations of lower agreeableness with MI were statistically significant only in the presence of met/met genotype; associations of higher neuroticism with MI and PCI were only statistically significant in the presence of the met/met genotype and the *met* allele, respectively. However, multiplicative interaction terms were only significant for BDNF val66met polymorphism and higher neuroticism on recurrent MI as an outcome.

## Discussion

The principal findings of this cohort study were that associations between personality traits and worse ACS prognosis were modified by the BDNF val66met polymorphism, in that observed associations were incrementally stronger and more likely to be statistically significant with increasing numbers of *met* alleles in the genotype. This modification gave rise to multiplicative interaction terms which were statistically significant mainly for composite MACE as the primary outcome and for MI or PCI as secondary outcomes. However, distributions by genotype of other exposure-outcome associations were broadly consistent. These findings suggest novel gene x environment interactions on long-term cardiac outcomes in ACS.

Considering the plausibility of the findings and their coherence with other research, although this type of investigation has not been previously carried out for ACS as a clinical syndrome, similar findings have been reported for other exposures and contexts. For example, the BDNF val66met polymorphism has been found to modify associations between childhood maltreatment and incident depression in children [34], and between recent stressful life events and incident depression in elders [35]. In those studies, the BDNF val66met polymorphism was not directly associated with depression, but the *met* allele conferred higher vulnerability to depression after stressful events, which were in keeping with the present findings. In addition, this polymorphism has also been found to modify the associations of severe medical illnesses including stroke and cancer with subsequent depression [36,37]. All these findings suggest that the BDNF val66met polymorphism at least has the potential to modify environmental influences on health and our findings presented here suggest that these appear to extend to long-term cardiac outcomes following ACS. Considering the international context, allele frequencies in an east Asian setting may have increased the opportunity to detect significant interactions. In particular, the present sample had higher *met* allele frequency (49%) compared to reports from Western samples (23~28%) [15,23], but similar to frequencies reported from other Asian populations [38].

Before drawing conclusions, several issues should be considered. First, the direct associations between BDNF val66met polymorphism and long-term cardiac outcomes were not significant. This is consistent with a previous report [13] although not with others reporting significant associations with the *met* [12] or *val* allele [14,15]. As discussed earlier gene-environment or gene-gene interactions might account for heterogeneous findings if allelic influences depend on the presence of given environmental stressors.

Second, the present study used the full FFM personality inventory, supporting both typological and dimensional approaches, and to our knowledge is the first to investigate associations between these constructs and long-term cardiac outcomes in ACS. Specifically, vulnerable personality type, and lower agreeableness and higher neuroticism dimensions predicted worse cardiac outcomes in the full analysed sample. Considering other typological personality constructs, type D personality is recognised to be associated with ACS prognosis [19,20]. The vulnerable personality type in the present study might well include the type D personality, as the concepts of 'negative affectivity’ and ‘social inhibition' in type D personality are similar in concept to the high neuroticism and low agreeableness from FFM personality dimensions [39]. With respect to individual personality dimensions, the neuroticism, agreeableness and conscientiousness dimensions of the FFM personality have been associated with increased cardiac morbidity and mortality in community populations [39,40], in keeping partly with the present findings. In summary, therefore, the present results replicated previous observations on personality and ACS prognosis, although the FFM offered a potentially more comprehensive approach.

Third, personality traits were associated with non-fatal MI and PCI outcomes but not with any mortality components of the composite MACE. These findings are not consistent with previous reports of associations between type D personality and increased cardiac events including mortality in patients with ACS [19,20]. Furthermore, other studies using FFM personality measures have also reported associations with mortality in community samples, although non-fatal outcomes were not specifically investigated [40,41]. Since, to our knowledge, there has been no previous study investigating associations of FFM personality with cardiac outcomes following ACS, it is difficult to draw a firm conclusion and replication is clearly required. However, in keeping with the present finding, the FFM personality was not associated with mortality in a meta-analysis in patients with cancer [42]. This may also be due to the fact that the mortality rates have been decreased in patients with ACS by the development of treatment technologies and reducing delay in patients’ seeking treatment^45^ and/or by selective survival in this particular cohort (who, by definition, had sustained a non-fatal acute cardiac event and had survived 3 months by the time they completed the personality inventory).

This study has several strengths. First, participants were recruited at baseline consecutively from all eligible patients with recent ACS, which increased the sample homogeneity. Second, measurement of psychiatric and cardiovascular characteristics used well-validated standard approaches. Third, the comprehensive personality model of FFM offered both typological and dimensional approaches, maximizing the potential for current and subsequent cross-testing of hypotheses. Fourth, a range of covariates were considered in the analyses. Fifth, cardiac outcomes were evaluated thoroughly in all baseline participants over a long follow-up period.

On the other hand, a number of limitations require consideration in drawing inferences. First, recruitment was carried out at a single site, which may limit the generalizability of the present findings, although a single center study has potential strengths in terms of consistency in evaluation and treatment. Second, personality traits were investigated 3 months after ACS as mentioned above and premorbid or informant-rated measures were not available. Third, there was a selection bias of the full baseline sample in terms of those agreeing to blood assays and undergoing the personality assessment. In particular, the analysed sample was younger and had more severe depressive and cardiac pathologies. Since, personality traits have been associated with both depression and cardiac diseases [43], it is possible that patients with vulnerable personality type were over-represented and therefore the strengths of associations between personality traits and cardiac outcomes increased, although these variables were included in the adjusted analyses. However, it is difficult to envisage how this could have influenced the gene-environment interactions of interest. Fourth, the small numbers experiencing individual MACE components (10.3% for cardiac death in particular) limited statistical power and potentially generalizability, although the incidence of composite MACE (42.4%) was sufficient.

## Conclusion

In patients with recent ACS, the pathogenic role of vulnerable personality on worse cardiac outcome varied according to BDNF val66met polymorphism. In clinical practice, personality evaluations with genetic testing of this polymorphism could potentially increase the predictability of cardiac prognosis, although this needs further empirical evaluation. Although gene-environment interactions in cardiovascular diseases have been received increasing research attention, only lifestyle and nutritional factors have been considered as environmental factors in ACS prognosis research up to now [5]. The present study could be used as a template for future study designs with a more biopsychosocial approach to predict long-term cardiac outcomes in ACS.

## Supporting information

S1 File Eligibility criteria for the DEPACS participants. (DOCX)

S2 File Ascertainment of the BDNF polymorphism. (DOCX)

S1 Fig Personality profile clusters according to Big Five Inventory z-scores (N = 611). (TIF)

S1 Table Effect of brain derived neurotrophic factor (BDNF) val66met polymorphism on major adverse cardiac outcomes (MACE) during the 5~12 years follow-up period after the index acute coronary syndrome in 611 patients with acute coronary syndrome. (DOCX)

S2 Table Effect of extraversion personality dimension on cumulative incidence (%) of major adverse cardiac events (MACE) by brain derived neurotrophic factor (BDNF) val66met polymorphism. (DOCX)

S3 Table Effect of agreeableness personality dimension on cumulative incidence (%) of major adverse cardiac events (MACE) by brain derived neurotrophic factor (BDNF) val66met polymorphism. (DOCX)

S4 Table Effect of conscientiousness personality dimension on cumulative incidence (%) of major adverse cardiac events (MACE) by brain derived neurotrophic factor (BDNF) val66met polymorphism. (DOCX)

S5 Table Effect of neuroticism personality dimension on cumulative incidence (%) of major sadverse cardiac events (MACE) by brain derived neurotrophic factor (BDNF) val66met polymorphism. (DOCX)

S6 Table Effect of openness personality dimension on cumulative incidence (%) of major adverse cardiac events (MACE) by brain derived neurotrophic factor (BDNF) val66met polymorphism. (DOCX)

## Abbreviations

ACS: Acute coronary syndrome
MACEs: major adverse cardiac events
MI: myocardial infarction
GEI: Gene-environment interactions
DEPACS: DEPression in ACS
BDNF: brain-derived neurotrophic factor
val66met: valine by methionine at codon 66
FFM: Five Factor Model
KAMIR: Korea Acute Myocardial Infarction Registry
BDI: Beck Depression Inventory
BMI: Body mass index
MINI: Mini International Neuropsychiatric Interview
CK-MB: Creatine kinase-MB
hs-CRP: C-reactive protein
DSM-IV: Diagnostic and Statistical Manual of Mental disorders, 4^th^ Edition
EKG: Elctrocardiography
LVEF: Left ventricular ejection fraction
PCR: Polymerase chain reaction
BFI: Big Five Inventory
PCI: percutaneous coronary intervention
