# Supplementary material for: Interaction between BDNF val66met polymorphism and personality on long-term cardiac outcomes in patients with acute coronary syndrome
Source: PLoS One. 2019 Dec 30;14(12):e0226802. doi: 10.1371/journal.pone.0226802 (PMC6936775; doi:10.1371/journal.pone.0226802)
Supplement: S1 File — (DOCX) [file pone.0226802.s001.docx]

**Eligibility Criteria for the DEPACS participants**

For the K-DEPACS study entry, inclusion criteria were as follows: i) aged 18~85 years; ii) confirmed ACS by investigation (the presence of ST-segment elevation MI was determined by >30 min of continuous chest pain, a new ST-segment elevation ≥2 mm on at least two contiguous electrocardiographic leads, and creatine kinase-MB more than three times normal; the presence of non-ST-segment elevation MI was diagnosed by chest pain and a positive cardiac biochemical marker without new ST-segment elevation; and the presence of unstable angina was determined by chest pain within the preceding 72 h with or without ST-T wave changes or positive cardiac biochemical markers); iii) ability to complete study questionnaires; iv) ability to understand the study objectives and sign informed consent. Exclusion criteria were: i) occurrence of ACS while hospitalized for another reason; ii) ACS developing less than 3 months after a coronary artery bypass graft procedure; iii) uncontrolled hypertension (systolic blood pressure (BP) >180mmHg or diastolic BP >100mmHg), the same criteria were used in the SADHART trial (Glassman et al. 2002); iv) resting heart rate <40/min; v) severe physical illnesses threatening life or interfering with the recovery from ACS; vi) persistent clinically significant laboratory abnormalities in complete blood cell counts, thyroid tests, renal function tests, and liver function tests.
