# Supplementary material for: Interaction between BDNF val66met polymorphism and personality on long-term cardiac outcomes in patients with acute coronary syndrome
Source: PLoS One. 2019 Dec 30;14(12):e0226802. doi: 10.1371/journal.pone.0226802 (PMC6936775; doi:10.1371/journal.pone.0226802)
Supplement: S2 File — (DOCX) [file pone.0226802.s002.docx]

**Ascertainment of the BDNF polymorphism**

DNA was extracted from venous blood using standard procedures. Polymerase chain reaction (PCR) and PCR-based restriction fragment length polymorphism assays were conducted. The forward and reverse primers had the sequences 5’-ACTCTGGAGAGCGTGAATGG-3’ and 5’‑ACTACTGAGCATCACCCTGGA-3’, respectively. The amplification conditions were pre-denaturation at 95°C for 5 min followed by 40 cycles of denaturation at 95°C for 30 s, 62°C for 30 s, and 72°C for 30 s, with post-elongation at 72°C for 5 min and a final maintenance step at 4°C. The PCR products were digested at 37°C with the corresponding restriction enzyme (*Eco*72I) and separated by gel electrophoresis to identify the 196G (*val*: 99- and 72-bp fragments) and 196A (*met*: 171-bp fragment) alleles. The genotype was categorized as val/val, val/met, or met/met.
