# Supplementary material for: Interaction between BDNF val66met polymorphism and personality on long-term cardiac outcomes in patients with acute coronary syndrome
Source: PLoS One. 2019 Dec 30;14(12):e0226802. doi: 10.1371/journal.pone.0226802 (PMC6936775; doi:10.1371/journal.pone.0226802)
Supplement: S1 Table — (DOCX) [file pone.0226802.s004.docx]

**S1 Table.** Effect of brain derived neurotrophic factor (BDNF) val66met polymorphism on major adverse cardiac outcomes (MACE) during the 5~12 years follow-up period after the index acute coronary syndrome in 611 patients with acute coronary syndrome.

| Events | N (%) of events by  BDNF val66met polymorphism | | | Unadjusted  HR (95% CI) | Adjusted^a^ | |
| --- | --- | --- | --- | --- | --- | --- |
|  | val/val  (N=151) | val/met  (N=324) | met/met  (N=136) |  | HR (95% CI) | P-value |
| MACE | 66 (43.7) | 136 (42.0) | 57 (41.9) | 0.96 (0.80-1.15) | 0.93 (0.78-1.12) | 0.469 |
| All-cause mortality | 27 (17.9) | 60 (18.5) | 19 (14.0) | 0.89 (0.67-1.18) | 0.90 (0.68-1.20) | 0.480 |
| Cardiac death | 15 (9.9) | 36 (11.1) | 12 (8.8) | 0.95 (0.66-1.36) | 0.92 (0.63-1.34) | 0.644 |
| Myocardial infarction | 17 (11.3) | 44 (13.6) | 21 (15.4) | 1.16 (0.85-1.60) | 1.13 (0.81-1.57) | 0.471 |
| Percutaneous coronary intervention | 25 (16.6) | 56 (17.3) | 22 (16.2) | 0.99 (0.75-1.31) | 0.95 (0.71-1.78) | 0.742 |

HR (95% CI) were calculated for one BDNF met allele increase using Cox proportional hazards models.

^a^Adjusted for age, gender, education, accommodation, Beck Depression Inventory scores, previous history of depression, hypertension, diabetes, hypercholesterolemia, obesity, smoking, past history of ACS, ACS diagnosis, Killip class, left ventricular ejection fraction, and serum levels on troponin I, creatine kinase-MB, and high sensitivity C-reactive protein at baseline.
