# Supplementary material for: Interaction between BDNF val66met polymorphism and personality on long-term cardiac outcomes in patients with acute coronary syndrome
Source: PLoS One. 2019 Dec 30;14(12):e0226802. doi: 10.1371/journal.pone.0226802 (PMC6936775; doi:10.1371/journal.pone.0226802)
Supplement: S5 Table — (DOCX) [file pone.0226802.s008.docx]

**S5 Table.** Effect of Neuroticism personality dimension on cumulative incidence (%) of major adverse cardiac events (MACE) by brain derived neurotrophic factor (BDNF) val66met polymorphism.

| Event | Patients group | Personality group | N patients | N (%)  events | Unadjusted  HR (95% CI) | Adusted | | P-value for interaction^b^ |
| --- | --- | --- | --- | --- | --- | --- | --- | --- |
|  |  |  |  |  |  | HR (95% CI) | P-value |  |
| MACE | All patients | Lower | 311 | 102 (32.8) | Ref | Ref | <0.001 |  |
|  |  | Higher | 300 | 157 (52.3) | 1.83 (1.42-2.34) | 1.70 (1.30-2.22) |  |  |
|  | **BDNF** | | | | | | | |
|  | *val/val* | Lower | 77 | 29 (37.7) | Ref | Ref | 0.318 | 0.026 |
|  |  | Higher | 74 | 37 (50.0) | 1.42 (0.87-2.31) | 1.38 (0.84-2.61) |  |  |
|  | *val/met* | Lower | 169 | 59 (34.9) | Ref | Ref | 0.024 |  |
|  |  | Higher | 155 | 77 (49.7) | 1.63 (1.16-2.29) | 1.52 (1.06-2.18) |  |  |
|  | *met/met* | Lower | 65 | 14 (21.5) | Ref | Ref | 0.001 |  |
|  |  | Higher | 71 | 43 (60.6) | 3.61 (1.97-6.63) | 3.50 (1.73-7.07) |  |  |
| All-cause  mortality | All patients | Lower | 311 | 47 (15.1) | Ref | Ref | 0.498 |  |
|  |  | Higher | 300 | 59 (19.7) | 1.35 (0.92-1.98) | 1.15 (0.76-1.74) |  |  |
|  | **BDNF** | | | | | | | |
|  | *val/val* | Lower | 77 | 14 (18.2) | Ref | Ref | 0.941 | 0.461 |
|  |  | Higher | 74 | 13 (17.6) | 0.96 (0.42-2.21) | 0.97 (0.39-2.38) |  |  |
|  | *val/met* | Lower | 169 | 27 (16.0) | Ref | Ref | 0.715 |  |
|  |  | Higher | 155 | 33 (21.3) | 1.42 (0.81-2.50) | 1.11 (0.64-1.92) |  |  |
|  | *met/met* | Lower | 65 | 6 (9.2) | Ref | Ref | 0.147 |  |
|  |  | Higher | 71 | 13 (18.3) | 2.20 (0.78-6.19) | 2.34 (0.74-7.41) |  |  |
| Cardiac death | All patients | Lower | 311 | 25 (8.0) | Ref | Ref | 0.286 |  |
|  |  | Higher | 300 | 38 (12.7) | 1.62 (0.98-2.68) | 1.34 (0.78-2.30) |  |  |
|  | **BDNF** | | | | | | | |
|  | *val/val* | Lower | 77 | 6 (7.8) | Ref | Ref | 0.561 | 0.331 |
|  |  | Higher | 74 | 6 (8.1) | 1.19 (0.67-3.51) | 0.60 (0.33-3.96) |  |  |
|  | *val/met* | Lower | 169 | 15 (8.9) | Ref | Ref | 0.532 |  |
|  |  | Higher | 155 | 21 (13.5) | 1.61 (0.80-3.25) | 1.26 (0.61-2.62) |  |  |
|  | *met/met* | Lower | 65 | 4 (6.2) | Ref | Ref | 0.103 |  |
|  |  | Higher | 71 | 11 (15.5) | 2.91 (1.28-12.97) | 2.22 (0.78-9.63) |  |  |
| Myocardial  infarction | All patients | Lower | 311 | 32 (10.3) | Ref | Ref | 0.091 |  |
|  |  | Higher | 300 | 50 (16.7) | 1.77 (1.13-2.75) | 1.50 (0.94-2.40) |  |  |
|  | **BDNF** | | | | | | | |
|  | *val/val* | Lower | 77 | 7 (9.1) | Ref | Ref | 0.465 | 0.027 |
|  |  | Higher | 74 | 10 (13.5) | 1.56 (0.56-4.35) | 1.28 (0.45-4.47) |  |  |
|  | *val/met* | Lower | 169 | 22 (13.0) | Ref | Ref | 0.860 |  |
|  |  | Higher | 155 | 22 (14.2) | 1.11 (0.59-2.09) | 1.06 (0.57-1.98) |  |  |
|  | *met/met* | Lower | 65 | 3 (4.6) | Ref | Ref | 0.011 |  |
|  |  | Higher | 71 | 18 (25.4) | 7.02 (1.96-25.15) | 5.88 (1.49-23.14) |  |  |
| Percutaneous  coronary  intervention | All patients | Lower | 311 | 36 (11.6) | Ref | Ref | 0.001 |  |
|  |  | Higher | 300 | 67 (22.3) | 2.11 (1.40-3.16) | 2.08 (1.36-3.19) |  |  |
|  | **BDNF** | | | | | | | |
|  | *val/val* | Lower | 77 | 9 (11.7) | Ref | Ref | 0.117 | 0.520 |
|  |  | Higher | 74 | 11 (21.6) | 2.08 (0.86-5.07) | 2.04 (0.78-5.94) |  |  |
|  | *val/met* | Lower | 169 | 22 (13.0) | Ref | Ref | 0.021 |  |
|  |  | Higher | 155 | 34 (21.9) | 1.88 (1.04-3.38) | 1.97 (1.11-3.52) |  |  |
|  | *met/met* | Lower | 65 | 5 (7.7) | Ref | Ref | 0.045 |  |
|  |  | Higher | 71 | 17 (23.9) | 3.78 (1.30-10.93) | 3.27 (1.05-10.71) |  |  |

HR (95% CI) were calculated using Cox proportional hazards models.

^a^Adjusted for age, gender, education, accommodation, Beck Depression Inventory scores, previous history of depression, hypertension, diabetes, hypercholesterolemia, obesity, smoking, past history of ACS, ACS diagnosis, Killip class, left ventricular ejection fraction, and serum levels on troponin I, creatine kinase-MB, and high sensitivity C-reactive protein at baseline.

^b^Multiplicative interaction terms between personality and BDNF val66met polymorphism on MACE in the same adjusted model.
